# Protocol for a systematic review and meta-analysis of pharmacological and non-pharmacological interventions for chronic pain management in chronic kidney disease

**DOI:** 10.1371/journal.pone.0343969

**Published:** 2026-03-03

**Authors:** Chi Peng Chan, Babaniji Omosule, Courtney Lightfoot, Ellesha A. Smith, Ffion Curtis, James O. Burton, Paul Gardner, Sarah Jasat, Sherna F. Adenwalla, Jyoti Baharani, Daniel S. March

**Affiliations:** 1 Department of Renal Medicine, University Hospitals Birmingham NHS Foundation Trust, Birmingham, United Kingdom; 2 Department of Population Health Sciences, University of Leicester, Leicester, United Kingdom; 3 NIHR Leicester Biomedical Research Centre, University Hospitals of Leicester NHS Trust, Leicester, United Kingdom; 4 Liverpool Reviews and Implementation Group (LRiG), University of Liverpool, Liverpool, United Kingdom; 5 Department of Cardiovascular Sciences, University of Leicester, Leicester, United Kingdom; 6 Department of Renal Medicine, University Hospitals of Leicester NHS Trust, Leicester, United Kingdom; 7 Renal and Pain Management Services West, Betsi Cadwaladr University Health Board, Bangor, United Kingdom; 8 Leicester Partnership for Kidney Health Research, University of Leicester, Leicester, United Kingdom; University of Yaounde I Faculty of Medicine and Biomedical Sciences: Universite de Yaounde I Faculte de Medecine et des Sciences Biomedicales, CAMEROON

## Abstract

**Background:**

Chronic pain affects up to 60% of people with chronic kidney disease (CKD), yet remains under-recognised and under-treated. Pain management in this population is complicated by altered drug pharmacokinetics, polypharmacy, and the potential nephrotoxicity of conventional analgesics. Despite the high prevalence and significant impact on quality of life, evidence-based guidance specific to pain management in CKD remains limited.

**Objectives:**

This systematic review aims to evaluate the effectiveness and safety of both pharmacological and non-pharmacological interventions in reducing chronic pain intensity among people with CKD on dialysis, not on dialysis, and kidney transplant recipients, across all stages of CKD.

**Methods:**

The primary outcome is the effectiveness of interventions in reducing chronic pain intensity as assessed by pain assessment tools. We will conduct a comprehensive search of MEDLINE, Embase, CINAHL, Web of Science, and ClinicalTrials.gov from their inception to the present date to identify studies for chronic pain management in people living with CKD. Study screening will be conducted independently by two reviewers. One reviewer will extract data from each study, with a second reviewer cross-checking for accuracy and completeness. Data will be extracted on study characteristics, participant demographics, intervention components, pain outcomes, and adverse events. The certainty of evidence will be evaluated independently by two reviewers using the GRADE approach. Where applicable, data will be combined in meta-analyses using random-effects models. Additionally, a network meta-analysis will be performed if enough studies are available.

**Expected results:**

This review will synthesise the current evidence for pain management strategies in CKD, by evaluating effectiveness of interventions among people receiving different renal replacement therapy modalities with varying pain and disease phenotypes. Findings will highlight the comparative effectiveness of various interventions while considering their safety profiles specific to the CKD context. The review will identify gaps in the literature and provide recommendations for clinical practice and future research.

**Significance:**

This review seeks to deliver a thorough evaluation of pain management strategies for people living with CKD. This systematic review is supported by the UK Kidney Association (UKKA), and findings will inform the upcoming UKKA guideline on symptoms management in people with CKD, alongside the other symptoms including itch, fatigue, and gastrointestinal symptoms. This review will aid clinicians in making well-informed decisions regarding pain management strategies, ensuring a balance between effectiveness and the specific risks associated with CKD.

## Introduction

Chronic pain affects up to 60% of people with chronic kidney disease (CKD) and up to 65% of those receiving dialysis therapy [1–3]. This strikingly high prevalence represents a significant but often overlooked burden within the CKD population. Despite its widespread occurrence and profound impact on quality of life, functional ability, and psychological wellbeing, pain in CKD remains under-recognised and under-treated in clinical practice [1,3–6].

Pain management in people with CKD presents unique challenges that extend beyond those encountered in the general population. Altered drug pharmacokinetics due to reduced renal clearance can lead to unpredictable drug levels and increased risk of adverse effects [7–9]. This physiological change is compounded by polypharmacy, which is common in people with CKD, creating complex drug interaction profiles [8,9]. Furthermore, many conventional analgesics, including non-steroidal anti-inflammatory drugs (NSAIDs), possess nephrotoxic properties that can potentially accelerate kidney function decline [10], presenting clinicians with difficult risk-benefit decisions.

The pain experience in CKD is heterogeneous, with musculoskeletal pain, abdominal pain, and neuropathic pain frequently reported [2,3,5]. Musculoskeletal pain often stems from CKD-mineral and bone disorder, secondary hyperparathyroidism, or vascular calcification processes unique to CKD [3,11,12]. Visceral pain may relate to polycystic kidney disease [3,5,13], uraemic gastropathy [14], or dialysis-associated peritoneal irritation [15]. Neuropathic symptoms, resulting from uraemic neuropathy or diabetes-associated complications, often manifest as burning, tingling sensations that significantly impair function and sleep [3,5,16,17]. Pain phenotypes may also evolve over time with CKD progression or treatment changes, further complicating management strategies [5].

Traditional pharmacological management approaches, particularly opioids, have been widely employed but are associated with significant mortality and morbidity in the CKD population [9,18–20]. Opioid accumulation due to reduced clearance can lead to respiratory depression, cognitive impairment, and heightened fall risk, while also potentially worsening uraemic symptoms such as pruritus and constipation [9,21]. The risk of dependence and addiction further complicates long-term use [9,19]. In addition, guidelines specific to opioid prescribing in the CKD population remain limited [6], contributing to wide variability in practice, and potential patient harm. Given the limitations of conventional analgesics, drug repurposing has gained attention as an alternative approach to chronic pain management. For example, preliminary evidence suggests that certain antihypertensive agents may offer analgesic benefits, although rigorous studies are required to confirm their efficacy and safety in people with chronic kidney disease [22]. In response to the challenges of conventional analgesics, there is also a growing interest in non-pharmacological interventions that may offer effective symptom relief with fewer risks [23]. A few examples include exercise programs tailored to CKD [24,25], complementary therapies (such as acupuncture, mindfulness meditation, and music therapy), cognitive behavioural therapy (CBT) [26], and pain coping skills training, which are increasingly being integrated into comprehensive pain management protocols with encouraging preliminary results [27,28].

Given the growing recognition of pain as a complex, multidimensional symptom that transcends physical discomfort, there is an urgent need for evidence-based, kidney-specific approaches to pain management [23]. Although the prevalence and adverse effects of different analgesic agents in CKD have been extensively investigated [9], data from interventional studies remain limited and fragmented, particularly for non-pharmacological treatments [5,9]. Most existing guidelines are extrapolated from non-CKD populations, raising concerns about their applicability and safety [5,9,29].

### Review question and objectives

This systematic review aims to evaluate the effectiveness of pharmacological and non-pharmacological interventions in reducing chronic pain intensity among people with CKD. We aim to synthesise findings across all CKD stages within three major cohorts, namely dialysis, non-dialysis (including pre-dialysis), and kidney transplant recipients, with a focus on effectiveness of interventions in reducing chronic pain intensity as assessed by pain assessment tools and the potential for integration into routine clinical practice [30,31].

Other objectives of this systematic review include:

- To collect serious adverse events reported in the primary studies associated with interventions demonstrating reported effectiveness;
- To evaluate the effectiveness of interventions in different pain phenotypes and specific renal diseases (e.g., polycystic kidney disease, calciphylaxis), if there are at least two or more studies available.

## Methods

### Protocol and registration

This systematic review will be conducted according to a pre-specified protocol registered with the International Prospective Register of Systematic Reviews (PROSPERO) (ID: CRD42024627482), in accordance with the PRISMA (Preferred Reporting Items for Systematic Reviews and Meta-Analyses) guidelines [32]. The protocol was collaboratively developed by all authors in accordance with the PRISMA-P (Preferred Reporting Items for Systematic Review and Meta-Analysis Protocols) Checklist [30,31] and finalised before the review commenced.

### Inclusion and exclusion criteria

The inclusion and exclusion criteria are summarised in Table 1.

**Table 1:** Summary of eligibility criteria by PICO framework and study type.

Domain	Include	Exclude	Example
**Population**	All adult and paediatric CKD patients with any one of the following: • CKD stages G3A-G5 (eGFR < 60 mL/min/1.73m²) [34]; • Undergoing dialysis (haemodialysis or peritoneal dialysis) [35]; • Kidney transplant recipients [35]; • ESKD patients receiving CKM [35]; • Post-dialysis withdrawal [35]; • Polycystic kidney disease with any eGFR; AND suffering from chronic pain, defined as pain lasting for more than three months [36,37].	• Patients suffering from acute or episodic pain, defined as pain lasting for less than three months. • Patients suffering from episodic pain related to procedures. • Patients suffering from acute pain related to operations.	Exclusion examples: • Muscle cramps during haemodialysis • AVF cannulation during haemodialysis • Post-operative analgesia in kidney transplant • AVF creation • Peritoneal catheter insertion
**Intervention**	Any pharmacological and non-pharmacological interventions for pain management in people with CKD [8,22].	Studies comparing pain outcomes in different renal replacement therapy modalities or techniques, without actual administration of an intervention for pain management.	
**Comparator**	• Any concurrent control group who is receiving usual care; OR • Any concurrent group who is receiving a different intervention intended for pain management; OR • Baseline group before intervention administration in before-and-after studies.	–	
**Outcomes**	• Primary outcome: The effectiveness of interventions in reducing chronic pain intensity (as assessed by pain assessment tools) in three different CKD cohorts, namely dialysis, non-dialysis, and kidney transplant recipients. This includes studies with a HRQoL scoring instrument which has also reported pain severity scores using equivalent pain assessment tools. • Secondary outcome: The serious adverse events reported in the primary studies associated with interventions demonstrating reported effectiveness [8,9].	–	
**Types of Study**	RCTs, non-RCTs, before-and after studies, and longitudinal cohort studies (including registry studies), which have administered an intervention to manage pain.	Animal studies, qualitative studies, case-control studies, cross-sectional studies, case reports and series, narrative literature reviews, clinical practical guidelines, editorials, and commentaries.	

CKD: Chronic kidney disease; eGFR: Estimated glomerular filtration rate; ESKD: End-stage kidney disease; CKM: Conservative kidney management; AVF: Arteriovenous fistula; HRQoL: Health-related quality of life; RCT: Randomised controlled trials.

### Outcomes

The primary outcome is the effectiveness of interventions for reducing chronic pain intensity, as assessed by pain assessment tools such as the single item 10-cm Visual Analogue Scale (VAS), 36-Item Short Form Survey (SF-36) [33], Kidney Disease Quality of Life Short Form (KDQOL-SF^TM^) [34], and other equivalent tools. Given the heterogeneity of chronic pain syndrome across different CKD cohorts, the primary outcome will be reported separately for each of the following three cohorts, namely dialysis, non-dialysis (including pre-dialysis), and kidney transplant recipients. Similarly, the primary outcome will be also be analysed separately for different groups of pharmacological and non-pharmacological interventions. The adverse and safety outcome linked to interventions demonstrating reported effectiveness in reducing chronic pain severity score will be summarised and presented as a secondary outcome.

### Information sources and search strategy

We will conduct comprehensive literature searches in the following electronic databases: MEDLINE (via Ovid), Embase, CINAHL, Web of Science Core Collection, and ClinicalTrials.gov (for ongoing and unpublished trials) [35]. The search will be supplemented with a Google Scholar search, with the first 200 titles screened for inclusion, and by reviewing reference lists of included studies and relevant systematic reviews or meta-analyses [35]. In addition, the authors of any relevant conference abstracts will also be contacted up to twice, two weeks apart, to obtain and include the raw data where possible. Any relevant preprints will be included, and handled in the same way as published studies. There will be no restrictions on publication date or minimum follow-up period. Non-English publications will also be considered, with inclusion decisions made individually during the full-text screening stage.

The search strategy has been developed using a combination of controlled vocabulary terms (MeSH terms) and free-text keywords related to chronic kidney disease, pain, and pain management interventions [35]. The search strategy has been adapted for use with other bibliographic databases in consultation with an information specialist. The full search strategies for all databases are provided in the supplementary material. Searches will be conducted from database inception to the present date.

### Study selection

All search results will be imported into Covidence (Veritas Health Innovation Ltd., Melbourne, Australia), a web-based screening and data extraction tool recommended by the Cochrane Collaboration [35,36]. After deduplication, titles and abstracts of all identified studies will be independently screened by two reviewers against the pre-defined inclusion criteria. Full-text articles will be retrieved for all potentially eligible studies and independently assessed by two reviewers. Disagreements at any stage will be resolved through discussion or consultation with a third reviewer when necessary [32,35]. The number of records identified, screened, included, and excluded at each stage will be summarised using a PRISMA 2020 flow diagram.

### Data extraction

Data extraction will be performed using an adapted Cochrane Data Extraction Template within Covidence. The Template for Intervention Description and Replication (TIDieR) checklist will be used to extract and report the intervention details [37]. One reviewer will extract data from each study, with a second reviewer cross-checking for accuracy and completeness. The following data will be extracted:

1. Study characteristics: Publication details, country, study design, duration, funding source
2. Participant characteristics: Demographics, CKD stage, renal replacement therapy status and modalities, co-morbidities, pain phenotype, pain severity, pain duration, pain assessment tools
3. Intervention details: Name, type (pharmacological or non-pharmacological) and group (e.g., exercise therapies and rehabilitation, complementary therapies, behaviour therapies, acupuncture therapy), rationale, providers, delivery method, type of locations, frequency/duration/intensity/dose, personalisation/titration/adaptation (if any), modifications (if any), and adherence [37]
4. Outcome data: Definition, measurement methods, timepoints, missing data, adverse events

Study authors will be contacted to verify key trial characteristics and obtain missing outcome data up to twice, two weeks apart, where possible.

### Quality assessment

Quality assessments will be conducted independently by two reviewers, and any disagreements will be resolved through discussion or arbitration by a third reviewer [38].

Study quality will be assessed using study design appropriate tools such as the National Institutes of Health (NIH) Study Quality Assessment Tools [38], or the mixed methods appraisal tool (MMAT) [39]. Using the NIH tool, studies will be classified as “good” (least risk of bias), “fair” (some bias but not sufficient to invalidate results), or “poor” (significant risk of bias lowering confidence in results). Using the MMAT tool, the criteria of the chosen category will be rated accordingly to inform the quality of the studies, and a sensitivity analysis may be performed at a later stage [39]. Prior to the review, calibration exercises will be conducted to ensure consistency in applying the quality assessment tools [35].

An overall study quality classification will be provided as appropriate. Tabulated quality appraisals, which show ratings across all criterion for these individual studies, will also be presented.

### Equity, diversity, and inclusion assessment

We will use the PRO EDI framework to extract participant equity data, including age, sex, gender, race, ethnicity, socioeconomic status, education level, and location [40,41]. The framework provides a structured approach to data extraction and reporting and will be used alongside the planned equality impact assessment and the GRADE assessment to inform judgments about the applicability of evidence, particularly for groups underrepresented in the included studies.

### Data synthesis and analysis

#### Primary outcomes.

For the primary outcomes, data analysis will be performed by using the netmeta package in the statistical software R [42]. Data from randomised controlled trials (RCTs) using comparable outcome measures will be pooled in meta-analyses, if appropriate, based on clinical and statistical homogeneity and availability of data [35]. For continuous outcomes, standardised mean differences (SMDs) with 95% confidence intervals will be used to summarise the average treatment effects using Hedges’ adjusted g [43,44]. If outcomes are measured using the same method across studies, average treatment effects will be summarised as mean differences. For dichotomous outcomes, risk ratios or odds ratios with 95% confidence intervals will be calculated using frequentist approach [35]. Data from non-randomised and observational studies will be pooled and synthesised separately in an exploratory analysis to minimise biases and confounders.

If studies are sufficiently homogeneous in design, comparison and outcome measures, two types of meta-analyses are planned:

1. Standard pairwise meta-analyses to compare each intervention independently to control using generic inverse variance random-effects models to incorporate the assumption that different studies assessed different but related treatment effects [35]. Hartung-Knapp small-sample adjustments will be applied to reflect uncertainty in the between-study heterogeneity [35].
2. Network meta-analyses (if sufficient studies are available) to determine the most efficacious intervention among multiple competing interventions [35]. Random-effects models will be applied, reflecting the assumption that studies estimate different but related treatment effects. The models will assume a common between-study variance for all treatment comparisons in the network. Multi-arm studies will be accounted for by reweighting all comparisons of each multi-arm study [45]. Results from the network meta-analysis will be presented as relative effect estimates (mean difference, SMD, risk ratios, or odds ratios) for each pair of interventions. Ranking probabilities will be calculated for each outcome [35]. For a global approach, design-by-treatment interaction models will be used to evaluate the assumption of consistency across the network [46]. The separating indirect from direct evidence (SIDE) approach will be used to assess any local inconsistency [47]. Transitivity will also be assessed by comparing the distributions of effect modifiers across comparisons. In the event of disconnected networks, analyses will be conducted either on the largest network, or separately for both networks. Findings from these analyses will be interpreted appropriately with caution.

**Assessment of heterogeneity:** To evaluate the presence of clinical heterogeneity, descriptive statistics for the studies and study population characteristics will be compared across the included studies within each pairwise meta-analysis comparison. Heterogeneity will be quantified using the I² statistic [35], this will be interpreted in combination with the respective between-study variance estimate. Between-study variance will be estimated using the DerSimonian and Laid method.

In cases of significant heterogeneity, potential causes will be explored through subgroup analyses or meta-regression (if more than ten studies) for prespecified variables. Potential sources of heterogeneity that will be considered include different pain phenotypes and any specific underlying renal diseases. If heterogeneity cannot be adequately explained, we will create a narrative synthesis from the included studies structured around the type of intervention, target population characteristics, and outcome measures. Data will be presented as text, tables, and figures using synthesis without meta-analysis (SWiM) reporting guidelines [48].

**Subgroup and sensitivity analyses:** If there are more than two relevant studies, exploratory subgroup analyses will also be performed, focusing on the following outcomes:

- Effectiveness of interventions in different pain phenotypes (e.g., musculoskeletal, abdominal, neuropathic) [8]
- Effectiveness of interventions in CKD populations with specific renal diseases (e.g., polycystic kidney disease, calciphylaxis) [13,49]

**Assessment of publication bias:** Publication and small study bias will be assessed when 10 or more studies are included in a meta-analysis using funnel plots for pairwise meta-analysis, comparison adjusted funnel plots for network meta-analysis, and Egger’s test for asymmetry [35].

#### Secondary outcomes.

Given the anticipated heterogeneity of outcome measures, the secondary outcome will only be extracted and analysed descriptively, with findings summarised and presented in tables and narrative forms. No formal statistical pooling will be conducted.

**Certainty of evidence:** We will assess the certainty of the evidence using the GRADE approach, as outlined in the GRADE handbook [50]. We will complete certainty assessments for primary outcome. The GRADE approach considers five criteria (study limitations, consistency of effect, imprecision, indirectness, publication bias) to assess the certainty of the body of evidence for each outcome. The certainty of evidence for each outcome will be rated as high, moderate, low, or very low. Two reviewers will independently appraise certainty ratings. Any disagreements will be resolved through discussion and consultation with a third review author where necessary. Findings will be presented as text and summary of findings tables.

### Patient and Public Involvement (PPI)

The UKKA guideline development process incorporates proportionate patient and public involvement through representation on the guideline committee, consistent with established standards for guideline development. This approach ensures that patient perspectives inform the overall direction and recommendations, providing reassurance that the guideline reflects both clinical expertise and lived experience, even where input is not sought for every specific subsection.

### Open science

To ensure data transparency and accessibility, all of the following will be deposited in an open repository (i.e., GitHub): (a) Full search exports, including a PRISMA 2020 flow diagram; (b) The data-extraction template; (c) The analysis scripts for any standard pairwise and network meta-analyses upon completion of the systematic review and meta-analysis.

## Summary

More than a decade ago, Wyne A and colleagues highlighted the lack of RCTs in relation to the efficacy of pharmacological agents, only managing to identify two papers on the efficacy of opioids in ESKD patients [51]. Although the adverse effects of different analgesic agents in CKD have been extensively investigated [9], there is by far no other studies comparing the effectiveness of different pharmacological and non-pharmacological interventions in the CKD population. There is also a lack of guidelines to advise on how to integrate these different interventions into routine care for people living with CKD.

To our best knowledge, this is the first systematic review and meta-analysis including the effectiveness of all pharmacological and non-pharmacological interventions used to manage chronic pain in people with CKD across all ages (both adults and paediatrics), stages, pain phenotypes, and renal replacement therapy modalities. The findings will focus on the comparative effectiveness of various interventions, while considering their safety profiles specific to the context of CKD. In addition, our study includes reported pain intensity scores on all relevant studies with a health-related quality of life (HRQoL) scoring instrument, hence providing extensive data and evidence on the impacts of various interventions on pain management in the CKD cohort.

The findings of this systematic review will be published in a peer-reviewed journal, where the results will be summarised and presented in lay language with appropriate infographics to ensure the data are accessible to a broader audience. More importantly, by providing a comprehensive assessment of pain management approaches in CKD using an inclusive study design and approach [52], this review will also inform evidence-based clinical guidelines, including the upcoming UKKA guideline on symptoms management in people with CKD, which will be readily available on the UKKA website as a section. This will ultimately translate into routine clinical pain management practices and enhance the quality of life of all people with CKD suffering from chronic pain. Furthermore, our systematic review will outline the ongoing evidence gaps in pain management in the CKD cohort, and potentially inform the direction of future local and global clinical trials and research in this area.

## Supporting information

S1 Data Search strategy. (PDF)

S1 File PRISMA-P checklist. (DOCX)

## References

[1] Davison SN, Jhangri GS. The impact of chronic pain on depression, sleep, and the desire to withdraw from dialysis in hemodialysis patients. J Pain Symptom Manage. 2005;30(5):465–73. doi: 10.1016/j.jpainsymman.2005.05.013 16310620

[2] Davison SN, Rathwell S, Ghosh S, George C, Pfister T, Dennett L. The prevalence and severity of chronic pain in patients with chronic kidney disease: a systematic review and meta-analysis. Can J Kidney Health Dis. 2021;8:2054358121993995. doi: 10.1177/2054358121993995 33680484 PMC7897838

[3] Lambourg E, Colvin L, Guthrie G, Murugan K, Lim M, Walker H, et al. The prevalence of pain among patients with chronic kidney disease using systematic review and meta-analysis. Kidney Int. 2021;100(3):636–49. doi: 10.1016/j.kint.2021.03.041 33940112

[4] Murtagh FEM, Addington-Hall J, Higginson IJ. The prevalence of symptoms in end-stage renal disease: a systematic review. Adv Chronic Kidney Dis. 2007;14(1):82–99. doi: 10.1053/j.ackd.2006.10.001 17200048

[5] Davison SN, Koncicki H, Brennan F. Pain in chronic kidney disease: a scoping review. Semin Dial. 2014;27(2):188–204. doi: 10.1111/sdi.12196 24517512

[6] Koncicki HM, Unruh M, Schell JO. Pain management in CKD: a guide for nephrology providers. Am J Kidney Dis. 2017;69(3):451–60. doi: 10.1053/j.ajkd.2016.08.039 27881247

[7] Pham PC, Khaing K, Sievers TM, Pham PM, Miller JM, Pham SV, et al. 2017 update on pain management in patients with chronic kidney disease. Clin Kidney J. 2017;10(5):688–97. doi: 10.1093/ckj/sfx080 28979781 PMC5622905

[8] Davison SN. Clinical pharmacology considerations in pain management in patients with advanced kidney failure. Clin J Am Soc Nephrol. 2019;14(6):917–31. doi: 10.2215/CJN.05180418 30833302 PMC6556722

[9] Lambourg E, Colvin L, Guthrie G, Walker H, Bell S. Analgesic use and associated adverse events in patients with chronic kidney disease: a systematic review and meta-analysis. Br J Anaesth. 2022;128(3):546–61. doi: 10.1016/j.bja.2021.08.035 34763813

[10] Nderitu P, Doos L, Jones PW, Davies SJ, Kadam UT. Non-steroidal anti-inflammatory drugs and chronic kidney disease progression: a systematic review. Fam Pract. 2013;30(3):247–55. doi: 10.1093/fampra/cms086 23302818

[11] Martin KJ, González EA. Metabolic bone disease in chronic kidney disease. J Am Soc Nephrol. 2007;18(3):875–85. doi: 10.1681/ASN.2006070771 17251386

[12] Haroon MM, Sayed S, Al-ghitany A, Ezzat H, Gheita TA. Rheumatic and musculoskeletal manifestations in renal hemodialysis patients. Int J Clin Rheumatol. 2018;13(5):263–9.

[13] Hogan MC, Norby SM. Evaluation and management of pain in autosomal dominant polycystic kidney disease. Adv Chronic Kidney Dis. 2010;17(3):e1–16. doi: 10.1053/j.ackd.2010.01.005 20439087 PMC4144785

[14] Turshudzhyan A, Inyangetor D. Uremic and post-transplant gastropathy in patients with chronic kidney disease and end-stage renal disease. Cureus. 2020;12(9):e10578. doi: 10.7759/cureus.10578 32983742 PMC7510509

[15] Hua S, Cao P, Zhang G, Li G, Jiang S, Xu Y, et al. The incidence of inflow and drain pain and associated risk factors for patients on peritoneal dialysis. BMC Nephrol. 2025;26(1):41. doi: 10.1186/s12882-025-03962-2 39863837 PMC11762884

[16] Krishnan AV, Kiernan MC. Uremic neuropathy: clinical features and new pathophysiological insights. Muscle Nerve. 2007;35(3):273–90. doi: 10.1002/mus.20713 17195171

[17] Mambelli E, Barrella M, Facchini MG, Mancini E, Sicuso C, Bainotti S, et al. The prevalence of peripheral neuropathy in hemodialysis patients. Clin Nephrol. 2012;77(6):468–75. doi: 10.5414/cn107188 22595389

[18] Kimmel PL, Fwu C-W, Abbott KC, Eggers AW, Kline PP, Eggers PW. Opioid prescription, morbidity, and mortality in United States dialysis patients. J Am Soc Nephrol. 2017;28(12):3658–70. doi: 10.1681/ASN.2017010098 28935654 PMC5698071

[19] Ishida JH, McCulloch CE, Steinman MA, Grimes BA, Johansen KL. Opioid analgesics and adverse outcomes among hemodialysis patients. Clin J Am Soc Nephrol. 2018;13(5):746–53. doi: 10.2215/CJN.09910917 29674340 PMC5969477

[20] Novick TK, Surapaneni A, Shin J-I, Alexander GC, Inker LA, Wright EA, et al. Associations of opioid prescriptions with death and hospitalization across the spectrum of estimated GFR. Clin J Am Soc Nephrol. 2019;14(11):1581–9. doi: 10.2215/CJN.00440119 31582462 PMC6832057

[21] Owsiany MT, Hawley CE, Triantafylidis LK, Paik JM. Opioid management in older adults with chronic kidney disease: a review. Am J Med. 2019;132(12):1386–93. doi: 10.1016/j.amjmed.2019.06.014 31295441 PMC6917891

[22] Du K, Li A, Zhang C-Y, Li S-M, Chen P. Repurposing antihypertensive drugs for pain disorders: a drug-target mendelian randomization study. Front Pharmacol. 2024;15:1448319. doi: 10.3389/fphar.2024.1448319 39268473 PMC11390634

[23] Lu E, Koncicki HM. Nonopioid approaches to pain management in chronic kidney disease. Semin Nephrol. 2021;41(1):54–67. doi: 10.1016/j.semnephrol.2021.02.006 33896474

[24] Johansen KL, Painter P. Exercise in individuals with CKD. Am J Kidney Dis. 2012;59(1):126–34. doi: 10.1053/j.ajkd.2011.10.008 22113127 PMC3242908

[25] Geneen LJ, Moore RA, Clarke C, Martin D, Colvin LA, Smith BH. Physical activity and exercise for chronic pain in adults: an overview of Cochrane Reviews. Cochrane Database Syst Rev. 2017;4(4):CD011279. doi: 10.1002/14651858.CD011279.pub3 28436583 PMC5461882

[26] Natale P, Palmer SC, Ruospo M, Saglimbene VM, Rabindranath KS, Strippoli GF. Psychosocial interventions for preventing and treating depression in dialysis patients. Cochrane Database Syst Rev. 2019;12(12):CD004542. doi: 10.1002/14651858.CD004542.pub3 31789430 PMC6886341

[27] Jhamb M, Steel JL, Yabes JG, Roumelioti M-E, Erickson S, Devaraj SM, et al. Effects of technology assisted stepped collaborative care intervention to improve symptoms in patients undergoing hemodialysis: the TĀCcare randomized clinical trial. JAMA Intern Med. 2023;183(8):795–805. doi: 10.1001/jamainternmed.2023.2215 37338898 PMC10282960

[28] Dember LM, Hsu JY, Mehrotra R, Cavanaugh KL, Kalim S, Charytan DM, et al. Pain coping skills training for patients receiving hemodialysis: the HOPE consortium randomized clinical trial. JAMA Intern Med. 2025;185(2):197–207. doi: 10.1001/jamainternmed.2024.7140 39786400 PMC11791705

[29] Barakzoy AS, Moss AH. Efficacy of the world health organization analgesic ladder to treat pain in end-stage renal disease. J Am Soc Nephrol. 2006;17(11):3198–203. doi: 10.1681/ASN.2006050477 16988057

[30] Moher D, Shamseer L, Clarke M, Ghersi D, Liberati A, Petticrew M, et al. Preferred reporting items for systematic review and meta-analysis protocols (PRISMA-P) 2015 statement. Syst Rev. 2015;4(1):1. doi: 10.1186/2046-4053-4-1 25554246 PMC4320440

[31] Shamseer L, Moher D, Clarke M, Ghersi D, Liberati A, Petticrew M, et al. Preferred reporting items for systematic review and meta-analysis protocols (PRISMA-P) 2015: elaboration and explanation. BMJ. 2015;350:g7647. doi: 10.1136/bmj.g7647 25555855

[32] Page MJ, McKenzie JE, Bossuyt PM, Boutron I, Hoffmann TC, Mulrow CD, et al. The PRISMA 2020 statement: an updated guideline for reporting systematic reviews. Int J Surg. 2021;88:105906. doi: 10.1016/j.ijsu.2021.105906 33789826

[33] Ware JE Jr, Sherbourne CD. The MOS 36-item short-form health survey (SF-36). I. Conceptual framework and item selection. Med Care. 1992;30(6):473–83. doi: 10.1097/00005650-199206000-00002 1593914

[34] Hays RD, Kallich J, Mapes D, Coons S, Amin N, Carter W, et al. Kidney disease quality of life short form (KDQOL-SF™), version 1.3: a manual for use and scoring. Santa Monica (CA): Rand; 1997. 7994 p.

[35] Higgins JPT, Thomas J, Chandler J, Cumpston M, Li T, Page MJ, et al. Cochrane handbook for systematic reviews of interventions version 6.5. Cochrane; 2024. Available from: www.cochrane.org/handbook

[36] Kellermeyer L, Harnke B, Knight S. Covidence and Rayyan. J Med Libr Assoc. 2018;106(4):580–3.

[37] Hoffmann TC, Glasziou PP, Boutron I, Milne R, Perera R, Moher D, et al. Better reporting of interventions: template for intervention description and replication (TIDieR) checklist and guide. BMJ. 2014;348:g1687. doi: 10.1136/bmj.g1687 24609605

[38] National Heart L, and Blood Institute. Study quality assessment tools. National Institutes of Health; 2021. Available from: https://www.nhlbi.nih.gov/health-topics/study-quality-assessment-tools

[39] Hong QN, Pluye P, Fàbregues S, Bartlett G, Boardman F, Cargo M, et al. Mixed methods appraisal tool (MMAT), version 2018. Registration of copyright. 2018;1148552(10):1–7.

[40] PRO EDI participant characteristics table; 2024. Available from: https://www.trialforge.org/trial-diversity/pro-edi-improving-how-equity-diversity-and-inclusion-is-handled-in-evidence-synthesis/

[41] Witham MD, Anderson E, Carroll C, Dark PM, Down K, Hall AS, et al. Developing a roadmap to improve trial delivery for under-served groups: results from a UK multi-stakeholder process. Trials. 2020;21(1):694. doi: 10.1186/s13063-020-04613-7 32738919 PMC7395975

[42] Balduzzi S, Rücker G, Schwarzer G. How to perform a meta-analysis with R: a practical tutorial. Evid Based Ment Health. 2019;22(4):153–60. doi: 10.1136/ebmental-2019-300117 31563865 PMC10231495

[43] Cohen J. Statistical power analysis for the behavioral sciences. Routledge; 2013.

[44] Hedges LV, Olkin I. Statistical methods for meta-analysis. Academic Press; 2014.

[45] Rücker G. Network meta-analysis, electrical networks and graph theory. Res Synth Methods. 2012;3(4):312–24. doi: 10.1002/jrsm.1058 26053424

[46] Jackson D, Boddington P, White IR. The design-by-treatment interaction model: a unifying framework for modelling loop inconsistency in network meta-analysis. Res Synth Methods. 2016;7(3):329–32. doi: 10.1002/jrsm.1188 26588593 PMC4946625

[47] Shih M-C, Tu Y-K. An evidence-splitting approach to evaluation of direct-indirect evidence inconsistency in network meta-analysis. Res Synth Methods. 2021;12(2):226–38. doi: 10.1002/jrsm.1480 33543575

[48] Campbell M, McKenzie JE, Sowden A, Katikireddi SV, Brennan SE, Ellis S, et al. Synthesis without meta-analysis (SWiM) in systematic reviews: reporting guideline. BMJ. 2020;368:l6890. doi: 10.1136/bmj.l6890 31948937 PMC7190266

[49] Chinnadurai R, Sinha S, Lowney AC, Miller M. Pain management in patients with end-stage renal disease and calciphylaxis- a survey of clinical practices among physicians. BMC Nephrol. 2020;21(1):403. doi: 10.1186/s12882-020-02067-2 32948131 PMC7501607

[50] Schünemann HBJ, Guyatt G, Oxman A, et al. GRADE handbook for grading quality of evidence and strength of recommendations. The GRADE Working Group; 2013. Available from: https://gdt.gradepro.org/app/handbook/handbook.html

[51] Wyne A, Rai R, Cuerden M, Clark WF, Suri RS. Opioid and benzodiazepine use in end-stage renal disease: a systematic review. Clin J Am Soc Nephrol. 2011;6(2):326–33. doi: 10.2215/CJN.04770610 21071517 PMC3052223

[52] Burton JO, Chilcot J, Fielding K, Frankel AH, Lakhani N, Nye P, et al. Best practice for the selection, design and implementation of UK Kidney Association guidelines: a modified Delphi consensus approach. BMJ Open. 2024;14(6):e085723. doi: 10.1136/bmjopen-2024-085723 38890135 PMC11191819

