# Supplementary material for: Protocol for a systematic review and meta-analysis of pharmacological and non-pharmacological interventions for chronic pain management in chronic kidney disease
Source: PLoS One. 2026 Mar 3;21(3):e0343969. doi: 10.1371/journal.pone.0343969 (PMC12956079; doi:10.1371/journal.pone.0343969)
Supplement: S1 Data — (PDF) [file pone.0343969.s001.pdf]

Pain Management in Chronic Kidney Disease: Systematic Review Search Strategy

MEDLINE (via Ovid):

1	Renal Insufficiency, Chronic/
2	Kidney Failure, Chronic/
3	(Kidney ADJ2 (end-stage OR end stage OR chronic OR conservative)).tw.
4	(Renal ADJ2 (end-stage OR end stage OR chronic OR conservative)).tw.
5	CKD.tw.
6	CRF.tw.
7	ESKD.tw.
8	ESRF.tw.
9	Renal Replacement Therapy/
10	exp Renal Dialysis/
11	Renal replacement therapy.tw.
12	Kidney replacement therapy.tw.
13	Dialysis.tw.
14	H?emodialysis.tw.
15	HD.tw.
16	Peritoneal dialysis.tw.
17	CAPD.tw.
18	APD.tw.
19	Kidney Transplantation/
20	Kidney transplant*.tw.
21	Renal transplant*.tw.
22	exp Polycystic Kidney Diseases/
23	Polycystic kidney.tw.
24	Calciphylaxis/
25	Calciphylaxis.tw.
26	1 OR 2 OR 3 OR 4 OR 5 OR 6 OR 7 OR 8 OR 9 OR 10 OR 11 OR 12 OR 13 OR 14 OR 15 OR 16 OR 17 OR 18 OR 19 OR 20 OR 21 OR 22 OR 23 OR 24 OR 25
27	Pain/
28	Chronic Pain/
29	Abdominal Pain/
30	Musculoskeletal Pain/
31	Neuralgia/
32	Back Pain/

33	Flank Pain/
34	Pelvic Pain/
35	Headache/
36	Fibromyalgia/
37	Pain.tw.
38	Neuralgia.tw.
39	Headache.tw.
40	Fibromyalgia.tw.
41	Quality of life.tw.
42	27 OR 28 OR 29 OR 30 OR 31 OR 32 OR 33 OR 34 OR 35 OR 36 OR 37 OR 38 OR 39 OR 40 OR 41
43	Pain Management/
44	(Pain ADJ2 (manag* OR relie* OR treat*)).tw.
45	43 OR 44
46	Analgesia/
47	exp Analgesics/
48	Analgesi*.tw.
49	Pain-killer*.tw.
50	Pain killer*.tw.
51	exp Analgesics, Opioid/
52	exp Anti-Inflammatory Agents, Non-Steroidal/
53	exp Acetaminophen/
54	exp Narcotic Antagonists/
55	exp Cyclohexanols/
56	exp Muscle Relaxants, Central/
57	exp gamma-Aminobutyric Acid/
58	exp Selective Serotonin Reuptake Inhibitors/
59	exp Antidepressive Agents, Tricyclic/
60	exp "Serotonin and Noradrenaline Reuptake Inhibitors"/
61	Lidocaine/
62	Capsaicin/
63	Cannabis/
64	Opioid*.tw.
65	Morphine.tw.
66	Oxycodone.tw.
67	Alfentanil.tw.

68	Fentanyl.tw.
69	Buprenorphine.tw.
70	Paracetamol.tw.
71	Tramadol.tw.
72	Pregabalin.tw.
73	Gabapentin.tw.
74	Amitriptyline.tw.
75	Codeine.tw.
76	Dihydrocodeine.tw.
77	NSAID*.tw.
78	Lidocaine.tw.
79	Capsaicin.tw.
80	Cannabis.tw.
81	Baclofen.tw.
82	46 OR 47 OR 48 OR 49 OR 50 OR 51 OR 52 OR 53 OR 54 OR 55 OR 56 OR 57 OR 58 OR 59 OR 60 OR 61 OR 62 OR 63 OR 64 OR 65 OR 66 OR 67 OR 68 OR 69 OR 70 OR 71 OR 72 OR 73 OR 74 OR 75 OR 76 OR 77 OR 78 OR 79 OR 80 OR 81
83	Rehabilitation/
84	exp Exercise Therapy/
85	exp Musculoskeletal Manipulations/
86	Occupational Therapy/
87	Hydrotherapy/
88	Aquatic Therapy/
89	exp Mind-Body Therapies/
90	Complementary Therapies/
91	Cupping Therapy/
92	exp Acupuncture Therapy/
93	Reflexotherapy/
94	Psychotherapy/
95	Behavior Therapy/
96	Cognitive Behavioral Therapy/
97	exp Medicine, Traditional/
98	Transcutaneous Electric Nerve Stimulation/
99	Rehabilitation.tw.
100	Exercise.tw.
101	Chiropractic.tw.

102	Osteopathic.tw.
103	Osteopathy.tw.
104	Acupressure.tw.
105	Massage.tw.
106	Occupational Therapy.tw.
107	Hydrotherapy.tw.
108	Cupping.tw.
109	Acupuncture.tw.
110	Reflexotherapy.tw.
111	Aromatherapy.tw.
112	Hypnosis.tw.
113	Autohypnosis.tw.
114	Meditation.tw.
115	Relaxation Technique.tw.
116	Yoga.tw.
117	Tai Ji.tw.
118	Tai Chi.tw.
119	Psychotherapy.tw.
120	Behavio?r Therapy.tw.
121	Cognitive Behavio?ral Therapy.tw.
122	CBT.tw.
123	Traditional Medicine.tw.
124	Traditional Chinese Medicine.tw.
125	TENS.tw.
126	Transcutaneous Electric*.tw.
127	83 OR 84 OR 85 OR 86 OR 87 OR 88 OR 89 OR 90 OR 91 OR 92 OR 93 OR 94 OR 95 OR 96 OR 97 OR 98 OR 99 OR 100 OR 101 OR 102 OR 103 OR 104 OR 105 OR 106 OR 107 OR 108 OR 109 OR 110 OR 111 OR 112 OR 113 OR 114 OR 115 OR 116 OR 117 OR 118 OR 119 OR 120 OR 121 OR 122 OR 123 OR 124 OR 125 OT 126
128	45 OR 82 OR 127
129	26 AND 42 AND 128

EMBASE (via OVID):

1	chronic kidney failure/
2	(Kidney ADJ2 (end-stage OR end stage OR chronic OR conservative)).tw.
3	(Renal ADJ2 (end-stage OR end stage OR chronic OR conservative)).tw.
4	CKD.tw.
5	CRF.tw.
6	ESKD.tw.
7	ESRF.tw.
8	renal replacement therapy/
9	hemodialysis/
10	peritoneal dialysis/
11	Renal replacement therapy.tw.
12	Kidney replacement therapy.tw.
13	Dialysis.tw.
14	H?emodialysis.tw.
15	HD.tw.
16	Peritoneal dialysis.tw.
17	CAPD.tw.
18	APD.tw.
19	kidney transplantation/
20	"Kidney transplant*".tw.
21	"Renal transplant*".tw.
22	kidney polycystic disease/
23	Polycystic kidney.tw.
24	Calciphylaxis.tw.
25	1 OR 2 OR 3 OR 4 OR 5 OR 6 OR 7 OR 8 OR 9 OR 10 OR 11 OR 12 OR 13 OR 14 OR 15 OR 16 OR 17 OR 18 OR 19 OR 20 OR 21 OR 22 OR 23 OR 24
26	pain/
27	chronic pain/
28	abdominal pain/
29	musculoskeletal pain/
30	neuralgia/
31	backache/
32	flank pain/
33	pelvic pain/
34	headache/

35	fibromyalgia/
36	Pain.tw.
37	Neuralgia.tw.
38	Headache.tw.
39	Fibromyalgia.tw.
40	Quality of life.tw.
41	26 OR 27 OR 28 OR 29 OR 30 OR 31 OR 32 OR 33 OR 34 OR 35 OR 36 OR 37 OR 38 OR 39 OR 40
42	(Pain ADJ2 (manag* OR relie* OR treat*)).tw.
43	"Analgesi*".tw.
44	"Pain-killer*".tw.
45	"Pain killer*".tw.
46	"Opioid*".tw.
47	Morphine.tw.
48	Oxycodone.tw.
49	Alfentanil.tw.
50	Fentanyl.tw.
51	Buprenorphine.tw.
52	Paracetamol.tw.
53	Tramadol.tw.
54	Pregabalin.tw.
55	Gabapentin.tw.
56	Amitriptyline.tw.
57	Codeine.tw.
58	Dihydrocodeine.tw.
59	"NSAID*".tw.
60	Lidocaine.tw.
61	Capsaicin.tw.
62	Cannabis.tw.
63	Baclofen.tw.
64	43 OR 44 OR 45 OR 46 OR 47 OR 48 OR 49 OR 50 OR 51 OR 52 OR 53 OR 54 OR 55 OR 56 OR 57 OR 58 OR 59 OR 60 OR 61 OR 62 OR 63
65	Rehabilitation.tw.
66	Exercise.tw.
67	Chiropractic.tw.
68	Osteopathic.tw.
69	Osteopathy.tw.

70	Acupressure.tw.
71	Massage.tw.
72	Occupational Therapy.tw.
73	Hydrotherapy.tw.
74	Cupping.tw.
75	Acupuncture.tw.
76	Reflexotherapy.tw.
77	Aromatherapy.tw.
78	Hypnosis.tw.
79	Autohypnosis.tw.
80	Meditation.tw.
81	Relaxation Technique.tw.
82	Yoga.tw.
83	Tai Ji.tw.
84	Tai Chi.tw.
85	Psychotherapy.tw.
86	Behavio?r Therapy.tw.
87	Cognitive Behavio?ral Therapy.tw.
88	CBT.tw.
89	Traditional Medicine.tw.
90	Traditional Chinese Medicine.tw.
91	TENS.tw.
92	"Transcutaneous Electric*".tw.
93	65 OR 66 OR 67 OR 68 OR 69 OR 70 OR 71 OR 72 OR 73 OR 74 OR 75 OR 76 OR 77 OR 78 OR 79 OR 80 OR 81 OR 82 OR 83 OR 84 OR 85 OR 86 OR 87 OR 88 OR 89 OR 90 OR 91 OR 92
94	42 OR 64 OR 93
95	25 AND 41 AND 94

CINAHL (EBSCO):

1	(MH "Renal Insufficiency, Chronic")
2	(MH "Kidney Failure, Chronic")
3	(TI "kidney" OR AB "kidney") N2 ((TI "end-stage" OR AB "end-stage") OR (TI "end stage" OR AB "end stage") OR (TI "chronic" OR AB "chronic") OR (TI "conservative" OR AB "conservative"))
4	(TI "renal" OR AB "renal") N2 ((TI "end-stage" OR AB "end-stage") OR (TI "end stage" OR AB "end stage") OR (TI "chronic" OR AB "chronic") OR (TI "conservative" OR AB "conservative"))
5	TI "CKD" OR AB "CKD"
6	TI "CRF" OR AB "CRF"
7	TI "ESKD" OR AB "ESKD"
8	TI "ESRF" OR AB "ESRF"
9	(MH "Renal Replacement Therapy")
10	(MH "Dialysis+")
11	TI "renal replacement therapy" OR AB "renal replacement therapy"
12	TI "kidney replacement therapy" OR AB "kidney replacement therapy"
13	TI "dialysis" OR AB "dialysis"
14	TI "h#emodialysis" OR AB "h#emodialysis"
15	TI "HD" OR AB "HD"
16	TI "peritoneal dialysis" OR AB "peritoneal dialysis"
17	TI "CAPD" OR AB "CAPD"
18	TI "APD" OR AB "APD"
19	(MH "Kidney Transplantation")
20	TI "kidney transplant*" OR AB "kidney transplant*"
21	TI "renal transplant*" OR AB "renal transplant*"
22	(MH "Kidney, Cystic+")
23	TI "polycystic kidney" OR AB "polycystic kidney"
24	(MH "Calciphylaxis")
25	TI "calciphylaxis" OR AB "calciphylaxis"
26	S1 OR S2 OR S3 OR S4 OR S5 OR S6 OR S7 OR S8 OR S9 OR S10 OR S11 OR S12 OR S13 OR S14 OR S15 OR S16 OR S17 OR S18 OR S19 OR S20 OR S21 OR S22 OR S23 OR S24 OR S25
27	(MH "Pain")
28	(MH "Chronic Pain")
29	(MH "Abdominal Pain")
30	(MH "Musculoskeletal Pain")
31	(MH "Neuralgia")

32	(MH "Back Pain")
33	(MH "Pelvic Pain")
34	(MH "Headache")
35	(MH "Fibromyalgia")
36	TI "pain" OR AB "pain"
37	TI "neuralgia" OR AB "neuralgia"
38	TI "headache" OR AB "headache"
39	TI "fibromyalgia" OR AB "fibromyalgia"
40	TI "quality of life" OR AB "quality of life"
41	S27 OR S28 OR S29 OR S30 OR S31 OR S32 OR S33 OR S34 OR S35 OR S36 OR S37 OR S38 OR S39 OR S40
42	(MH "Pain Management")
43	(TI "pain" OR AB "pain") N2 ((TI "manag*" OR AB "manag*") OR (TI "relie*" OR AB "relie*") OR (TI "treat*" OR AB "treat*"))
44	S42 OR S43
45	(MH "Analgesia")
46	(MH "Analgesics")
47	TI "analgesi*" OR AB "analgesi*"
48	TI "pain-killer*" OR AB "pain-killer*"
49	TI "pain killer*" OR AB "pain killer*"
50	(MH "Analgesics, Opioid+")
51	(MH "Antiinflammatory Agents, Non-Steroidal+")
52	(MH "Acetaminophen")
53	(MH "Narcotic Antagonists+")
54	(MH "Tramadol")
55	(MH "Muscle Relaxants, Central+")
56	(MH "Gabapentin")
57	(MH "Pregabalin")
58	(MH "Serotonin Uptake Inhibitors+")
59	(MH "Antidepressive Agents, Tricyclic+")
61	(MH "Lidocaine")
62	(MH "Capsaicin")
63	(MH "Cannabis+")
64	TI "opioid*" OR AB "opioid*"
65	TI "morphine" OR AB "morphine"
66	TI "oxycodone" OR AB "oxycodone"
67	TI "alfentanil" OR AB "alfentanil"

68	TI "fentanyl" OR AB "fentanyl"
69	TI "buprenorphine" OR AB "buprenorphine"
70	TI "paracetamol" OR AB "paracetamol"
71	TI "tramadol" OR AB "tramadol"
72	TI "pregabalin" OR AB "pregabalin"
73	TI "gabapentin" OR AB "gabapentin"
74	TI "amitriptyline" OR AB "amitriptyline"
75	TI "codeine" OR AB "codeine"
76	TI "dihydrocodeine" OR AB "dihydrocodeine"
77	TI "NSAID*" OR AB "NSAID*"
78	TI "lidocaine" OR AB "lidocaine"
79	TI "capsaicin" OR AB "capsaicin"
80	TI "cannabis" OR AB "cannabis"
81	TI "baclofen" OR AB "baclofen"
82	S45 OR S46 OR S47 OR S48 OR S49 OR S50 OR S51 OR S52 OR S53 OR S54 OR S55 OR S56 OR S57 OR S58 OR S59 OR S60 OR S61 OR S62 OR S63 OR S64 OR S65 OR S66 OR S67 OR S68 OR S69 OR S70 OR S71 OR S72 OR S73 OR S74 OR S75 OR S76 OR S77 OR S78 OR S79 OR S80 OR S81
83	(MH "Rehabilitation")
84	(MH "Therapeutic Exercise+")
85	(MH "Chiropractic+")
86	(MH "Osteopathy+")
87	(MH "Applied Kinesiology")
88	(MH "Occupational Therapy")
89	(MH "Hydrotherapy")
90	(MH "Aquatic Exercises")
91	(MH "Mind Body Techniques+")
92	(MH "Alternative Therapies+")
93	(MH "Cupping Therapy")
94	(MH "Acupuncture+")
95	(MH "Massage+")
96	(MH "Psychotherapy+")
97	(MH "Behavior Therapy")
98	(MH "Cognitive Therapy")
99	(MH "Medicine, Traditional+")
100	(MH "Transcutaneous Electric Nerve Stimulation")
101	TI "rehabilitation" OR AB "rehabilitation"

102	TI "exercise" OR AB "exercise"
103	TI "chiropractic" OR AB "chiropractic"
104	TI "osteopathic" OR AB "osteopathic"
105	TI "osteopathy" OR AB "osteopathy"
106	TI "acupressure" OR AB "acupressure"
107	TI "massage" OR AB "massage"
108	TI "occupational therapy" OR AB "occupational therapy"
109	TI "hydrotherapy" OR AB "hydrotherapy"
110	TI "cupping" OR AB "cupping"
111	TI "acupuncture" OR AB "acupuncture"
112	TI "reflexotherapy" OR AB "reflexotherapy"
113	TI "aromatherapy" OR AB "aromatherapy"
114	TI "hypnosis" OR AB "hypnosis"
115	TI "autohypnosis" OR AB "autohypnosis"
116	TI "meditation" OR AB "meditation"
117	TI "relaxation technique" OR AB "relaxation technique"
118	TI "yoga" OR AB "yoga"
119	TI "tai ji" OR AB "tai ji"
120	TI "tai chi" OR AB "tai chi"
121	TI "psychotherapy" OR AB "psychotherapy"
122	TI "behavio#r therapy" OR AB "behavio#r therapy"
123	TI "cognitive behavio#ral therapy" OR AB "cognitive behavio#ral therapy"
124	TI "CBT" OR AB "CBT"
125	TI "traditional medicine" OR AB "traditional medicine"
126	TI "traditional Chinese medicine" OR AB "traditional Chinese medicine"
127	TI "TENS" OR AB "TENS"
128	TI "transcutaneous electric*" OR AB "transcutaneous electric*"
129	S83 OR S84 OR S85 OR S86 OR S87 OR S88 OR S89 OR S90 OR S91 OR S92 OR S93 OR S94 OR S95 OR S96 OR S97 OR S98 OR S99 OR S100 OR S101 OR S102 OR S103 OR S104 OR S105 OR S106 OR S107 OR S108 OR S109 OR S110 OR S111 OR S112 OR S113 OR S114 OR S115 OR S116 OR S117 OR S118 OR S119 OR S120 OR S121 OR S122 OR S123 OR S124 OR S125 OR S126 OR S127 OR S128
130	S44 OR S82 OR S129
131	S26 AND S41 AND S130

Web of Science Core Collection

1	((((((((((((((((((((((((((((((((((TS = (“chronic renal insufficiency”)) OR TS = (“chronic kidney failure”)) OR TS = (“chronic kidney disease”)) OR TS = (“end-stage kidney disease”)) OR TS = (“end stage kidney disease”)) OR TS = (“end-stage kidney failure”)) OR TS = (“end stage kidney failure”)) OR TS = (“conservative kidney management”)) OR TS = (“chronic renal failure”)) OR TS = (“chronic renal disease”)) OR TS = (“end-stage renal disease”)) OR TS = (“end stage renal disease”)) OR TS = (“end-stage renal failure”)) OR TS = (“end stage renal failure”)) OR TS = (“conservative renal management”)) OR TS = (“CKD”)) OR TS = (“CRF”)) OR TS = (“ESKD”)) OR TS = (“ESRF”)) OR TS = (“renal replacement therapy”)) OR TS = (“kidney replacement therapy”)) OR TS = (“dialysis”)) OR TS = (“haemodialysis”)) OR TS = (“hemodialysis”)) OR TS = (“HD”)) OR TS = (“peritoneal dialysis”)) OR TS = (“CAPD”)) OR TS = (“APD”)) OR TS = (“kidney transplantation”)) OR TS = (“kidney transplant”)) OR TS = (“renal transplantation”)) OR TS = (“renal transplant”)) OR TS = (“polycystic kidney disease”)) OR TS = (“calciphylaxis”))
2	(((((((((((TS = (“pain”)) OR TS = (“chronic pain”)) OR TS = (abdominal pain”)) OR TS = (“musculoskeletal pain”)) OR TS = (“neuralgia”)) OR TS = (“back pain”)) OR TS = (“flank pain”)) OR TS = (“pelvic pain”)) OR TS = (“headache”)) OR TS = (“fibromyalgia”)) OR TS = (“quality of life”))
3	(((TS = (“pain management”)) OR TS = (“pain relief”)) OR TS = (“pain treatment”)) OR TS = (“analgesia”)) OR TS = (“analgesic”)) OR TS = (“analgesics”)) OR TS = (“pain-killer”)) OR TS = (“pain-killers”)) OR TS = (“pain killer”)) OR TS = (“pain killers”)) OR TS = (“opioid”)) OR TS = (“codeine”)) OR TS = (“dihydrocodeine”)) OR TS = (“morphine”)) OR TS = (“oxycodone”)) OR TS = (“fentanyl”)) OR TS = (“alfentanil”)) OR TS = (“buprenorphine”)) OR TS = (“tramadol”)) OR TS = (“paracetamol”)) OR TS = (“acetaminophen”)) OR TS = (“non-steroidal anti-inflammatory drug)) OR TS = (“NSAID”) OR TS = (“pregabalin”)) OR TS = (“gabapentin”)) OR TS = (“amitriptyline”)) OR TS = (“lidocaine”)) OR TS = (“capsaicin”)) OR TS = (“cannabis”)) OR TS = (“baclofen”)) OR TS = (“rehabilitation”)) OR TS = (“exercise”)) OR TS = (“chiropractic”)) OR TS = (“osteopathic”)) OR TS = (“osteopathy”)) OR TS = (“acupressure”)) OR TS = (“occupational therapy”)) OR TS = (“hydrotherapy”)) OR TS = (“aquatic therapy”)) OR TS = (“mind-body therapies”)) OR TS = (“cupping”)) OR TS = (“acupuncture”)) OR TS = (“reflexotherapy”)) OR TS = (“massage”)) OR TS = (“psychotherapy”)) OR TS = (“behavior therapy”)) OR TS = (“behaviour therapy”)) OR TS = (“cognitive behavioral therapy”)) OR TS = (“cognitive behavioural therapy”)) OR TS = (“CBT”)) OR TS = (“traditional medicine”)) OR TS = (“traditional Chinese medicine”)) OR TS = (“transcutaneous electric nerve stimulation”)) OR TS = (“TENS”)) OR TS = (“aromatherapy”)) OR TS = (“hypnosis”)) OR TS = (“autohypnosis”)) OR TS = (“relaxation technique”)) OR TS = (“yoga”)) OR TS = (“Tai Ji”)) OR TS = (“Tai Chi”))
4	1 AND 2 AND 3

ClinicalTrials.gov: 16

Condition/disease: Chronic Kidney Disease
Other terms: Pain, Chronic
Study type: All studies
Study results: All studies
Intervention/treatment: Pain Management

Google Scholar: First 200

"Chronic kidney disease" + "Pain management"
